# Classification of Asphalt Pavement Cracks Using Laplacian Pyramid-Based Image Processing and a Hybrid Computational Approach

**DOI:** 10.1155/2018/1312787

**Published:** 2018-10-01

**Authors:** Nhat-Duc Hoang

**Affiliations:** Faculty of Civil Engineering, Institute of Research and Development, Duy Tan University, P809-03 Quang Trung, Da Nang, Vietnam

## Abstract

To improve the efficiency of the periodic surveys of the asphalt pavement condition, this study puts forward an intelligent method for automating the classification of pavement crack patterns. The new approach relies on image processing techniques and computational intelligence algorithms. The image processing techniques of Laplacian pyramid and projection integral are employed to extract numerical features from digital images. Least squares support vector machine (LSSVM) and Differential Flower Pollination (DFP) are the two computational intelligence algorithms that are employed to construct the crack classification model based on the extracted features. LSSVM is employed for data classification. In addition, the model construction phase of LSSVM requires a proper setting of the regularization and kernel function parameters. This study relies on DFP to fine-tune these two parameters of LSSVM. A dataset consisting of 500 image samples and five class labels of alligator crack, diagonal crack, longitudinal crack, no crack, and transverse crack has been collected to train and verify the established approach. The experimental results show that the Laplacian pyramid is really helpful to enhance the pavement images and reveal the crack patterns. Moreover, the hybridization of LSSVM and DFP, named as DFP-LSSVM, used with the Laplacian pyramid at the level 4 can help us to achieve the highest classification accuracy rate of 93.04%. Thus, the new hybrid approach of DFP-LSSVM is a promising tool to assist transportation agencies in the task of pavement condition surveying.

## 1. Introduction

Asphalt pavement is an important part of the national transportation network. Surveying tasks performed by local transportation agencies are very crucial for collecting the current condition of asphalt pavement. Based on the collected pavement condition, road distresses can be detected, classified, evaluated, and documented for determining the maintenance methods and priority. Since transportation agencies around the world are struggling to allocate their limiting resources to a large number of deteriorating road sections, acquiring the condition of road pavement in a timely manner has become indispensible to correctly rank rehabilitation projects which are currently competing for budget.

According to a recent report of the Central Intelligence Agency, the total road network length of the world has reached the figure of 64,285,009 km. Particularly, in Vietnam, based on the statistics done by the General Statistics Office of Viet Nam 2010 [1], the total length of asphalt pavement road has amounted to 93,535 km. Needless to say, this large number of road sections necessitates a huge cost for the rehabilitation operations yearly and makes road maintenance an increasingly challenging task.

As pointed out by Al-Suleiman et al. [2], the aging and downgrading processes of pavements are inevitable and can be caused by surface fatigue, shear developing in the subgrade, subbase, base, or surface layers. Among the various types of pavement distress, cracking is one of the most easily identified forms of defect. In pavement maintenance practice, cracks are often considered as the most important indicator of pavement deterioration. It is because cracks are generally associated with the structural integrity of the asphalt pavements and directly affect their serviceability [3]. Thus, timely and correct identification of cracks in pavement structure is crucial for appraising the structure condition, establishing proper rehabilitation approaches, and recovering the acceptable quality of pavement structures.

In developing countries including Vietnam, visual inspection and manual data processing carried out by human inspectors are the prevailing method of pavement survey. Although this approach can deliver accurate assessments, it is only appropriate for evaluating a small number of road sections. It is because manual surveying is notorious for low productivity as well as inconsistent results due to subjective judgments of road inspectors [4]. Accordingly, methods for automating this tedious surveying works, and expediting the data processing tasks is an urgent demand of transportation agencies in many countries especially Vietnam. Such methods not only help us to improve the productivity but also help us to ensure the consistency of evaluation outcomes. The assessment results obtained quickly by these automated methods can significantly help us to allocate the government budget reasonably to competing pavement maintenance projects.

Due to the aforementioned motivations, various studies have recently proposed intelligent models for automating the pavement survey process. In these models, signals acquired by two-dimensional digital images are the main objects under analysis. The reason is that pavement cracks can be generally recognized via the pixel intensities and the shape of cracks. Nevertheless, previous works have demonstrated that image-based crack detection is not an easy task. It is because pavement images often have complex background texture and diverse patterns of cracks. The images also feature heterogeneous pixel intensity and suffer from inconsistency of illumination condition [4].

To cope with such challenges, a large number of studies have resorted to advanced image processing techniques to enhance and extract numerical features which are useful for crack recognition from the digital images. Mahler et al. [5], Kirschke, Velinsky [6], and Cheng et al. [7] established intelligent methods relied on the image intensity thresholding for recognizing cracks; these methods are based on the assumption that crack objects usually have lower intensity values than those of the regions in the background. A simplified method for crack category realization based on the concept of crack type index was proposed by Lee and Kim [8]; this approach is also based on image thresholding techniques, and information computed from neighboring pixels is used to distinguish the state of cracks from no cracks.

Besides the widely used image thresholding methods, the beamlet transform [9], predesigned image filtering [10], the Gabor filter [11], weighted neighborhood segmentation [12], wavelet-morphology-based detection [13], fuzzy Hough transform [14], steerable matched filtering [15], probabilistic generative model [4], and optimized minimal path selection [16] have been investigated by various scholars. Deep learning approaches [17, 18] which automate the feature extraction process have also been proposed. Nevertheless, these complex models are also computational intensive and require capable machine to perform the computing tasks. For the case of deep learning, a large number of image samples are needed to construct robust pavement classification models. Moreover, the training process of deep learning-based models is also very time-consuming.

Recent literature review has shown a notable trend of combining advanced image processing techniques and computational intelligence approaches. The computational intelligence models are then employed for crack detection and classification based on the features extracted by image processing techniques. These hybrid models are capable of both detecting the appearance of cracks and classifying cracks into predefined patterns.

A study that compared the performance of multilayer perceptron neural network, genetic algorithms, and self-organizing maps in pavement crack classification was accomplished by Rababaah [19]. Mokhtari et al. [20] relied on neural network models for pavement crack detection; this study found that neural network models demonstrated better prediction performances than those of other learning strategies including decision tree and *k*-nearest neighbor. Banharnsakun [21] combined the artificial bee colony and the artificial neural network for pavement surface distress categorization; the former computational method was employed in the phase of image segmentation; the image classification was performed by the latter approach. Cubero-Fernandez et al. [22] recently employed various image processing techniques of logarithmic transformation, bilateral filter, Canny algorithm, and morphological filter to extract features from digital images; the extracted features were then analyzed by a classification tree.

Fujita et al. [23] and Wang et al. [24] relied on the support vector machine to establish crack classification models. An intelligent method based on steerable filter, support vector machine, neural network, and random forest has been described in the study of Hoang and Nguyen [25]. Recent reviewing works [26, 27] pointed out an increasing trend of applying a hybridization of image processing and computational approaches in pavement crack detection and categorization. Due to the criticality of the problem at hand, investigating other image processing and computational intelligence methods to develop models for pavement crack classification can greatly contribute to the body of knowledge and provide practitioners with other capable alternatives. This study first proposes to employ the image processing technique of Laplacian pyramid in the phase of feature extraction. Laplacian pyramid, as a pyramid-based image process, can be highly useful for representing and analyzing images to facilitate the task of recognizing pavement cracks. Furthermore, a hybrid model of least squares support vector machine and Differential Flower Pollination is then used to construct a pavement crack classification model based on the numerical features derived from the Laplacian pyramid.

The rest of the study is organized as follows: Section 2 describes the research methodology, followed by the section of image acquisition.  Section 3 depicts the structure of the proposed computational intelligence, followed by the report of experimental result (Section 4). The final section summarizes this research work with several conclusions.

## 2. Research Methodology

### 2.1. Image Processing Techniques

#### 2.1.1. Laplacian Pyramid

The pyramid-based image process is a popular method for solving basic problems in image analysis or manipulation including data compression and shape analysis [28–30]. This method has recently attracted many scholars in solving various tasks that involve image processing such as automatic segmentation [31], texture classification [32, 33], nonlinear image registration [34], and remote sensing [35].

Essentially, the image pyramid is a data structure devised to facilitate efficient scaled convolution by means of decreased image representation. This method includes a sequence of versions of an original image in which both sample density and resolution are reduced in regular steps [36]. In the case of the Gaussian pyramid, the next pyramid level is attained by low-pass filtering and subsampling the previous pyramid with a factor of two. In general, the levels of the pyramid are obtained iteratively as follows [36]:

(1) $$\begin{array}{c} \text{for}\;0<\text{l}<\text{N}:\;\;G\left(i,j\right)\underset{m}{\sum }\underset{n}{\sum }w\left(m,n\right)G_{l-1}\left(2i+m,2j+n\right), \end{array}$$

where the original image is denoted as *G*_0_. The next pyramid levels are denoted as *G*_1_, *G*_2_,…, *G*_*N*_.

In the task of pavement crack recognition, bandpass-filtered images are highly useful for revealing the patterns of cracks. The bandpass-filtered images of pavement images can be computed by subtracting each Gaussian pyramid level from the next lower level in the pyramid. Since the two consecutive levels of pyramid are different in the density of the sample, it is required to perform an image expansion process for the next lower level of the pyramid as follows [36]:

(2) $$\begin{array}{c} G\left(i,j\right)=4\underset{m}{\sum }\underset{n}{\sum }G_{l,k-l}\left(\frac{2i+m}{2},\frac{2j+n}{2}\right), \end{array}$$

with *G*_*l,0*_ = *G*_*l*_ and *k* > 0.

Accordingly, the next level of the bandpass pyramid (*L*_1_,…, *L*_*N*_) can be computed as follows:

(3) $$\begin{array}{c} L_{l}=G_{l}-G_{l+1,l}. \end{array}$$

**Figure 1:**
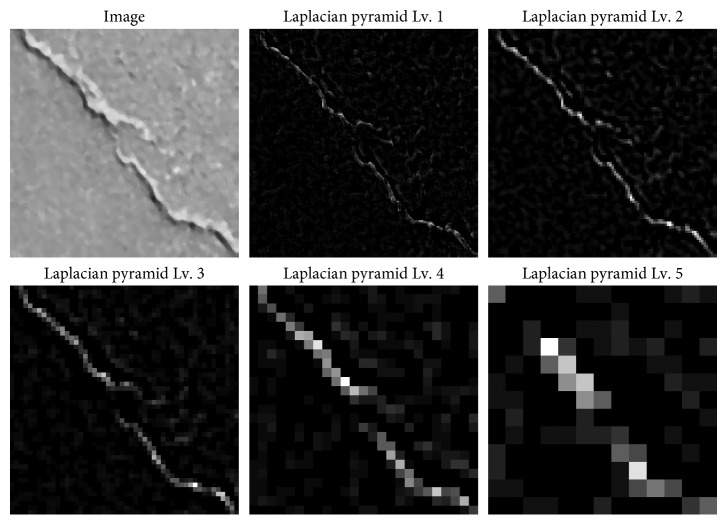
Demonstrations of the Laplacian pyramid with 5 levels.

It is noted that each value of this bandpass pyramid could be attained by convolving a difference of two Gaussians with the original image. This bandpass pyramid is very similar to the Laplacian operation. Thus, the bandpass pyramid can be regarded as the Laplacian pyramid. The responses of the the Laplacian pyramid applied for a pavement image are presented in Figure 1. It is proper to note that the Laplacian pyramid provides a complete image representation. It is because the steps employed to construct the pyramid can be performed in a reversed order to obtain the original image as follows [36]:

(4) $$\begin{array}{c} G_{0}=\sum L_{l,l}. \end{array}$$

#### 2.1.2. Projection Integrals

Projection integral (PI) is a widely employed method for in shape and texture classification [37]. This method has been shown to be highly applicable in the field of pavement defect detection and classification [22, 25, 38]. The first step of the PI approach is to convert an original RGB image into a gray-scale one. Based on the gray-scale image, the average value of the gray intensity at each location of the image along an axis is computed to obtain a projection integral (PI).

PIs along the horizontal and vertical axes are denoted as HPI and VPI, respectively. They are computed as follows:

(5) $$\begin{array}{c} \text{HPI}\left(y\right)=\underset{i\in x_{y}}{\sum }I\left(i,y\right),\\ \text{VPI}\left(x\right)=\underset{j\in y_{x}}{\sum }I\left(x,j\right), \end{array}$$

where HPI and VPI denote the horizontal and vertical PIs, respectively. *x*_*y*_ and *y*_*x*_ are the set of horizontal pixels at the vertical pixel *y* and the set of vertical pixels at the horizontal pixel *x* of an image *I*(*x*, *y*), respectively.

**Figure 2:**
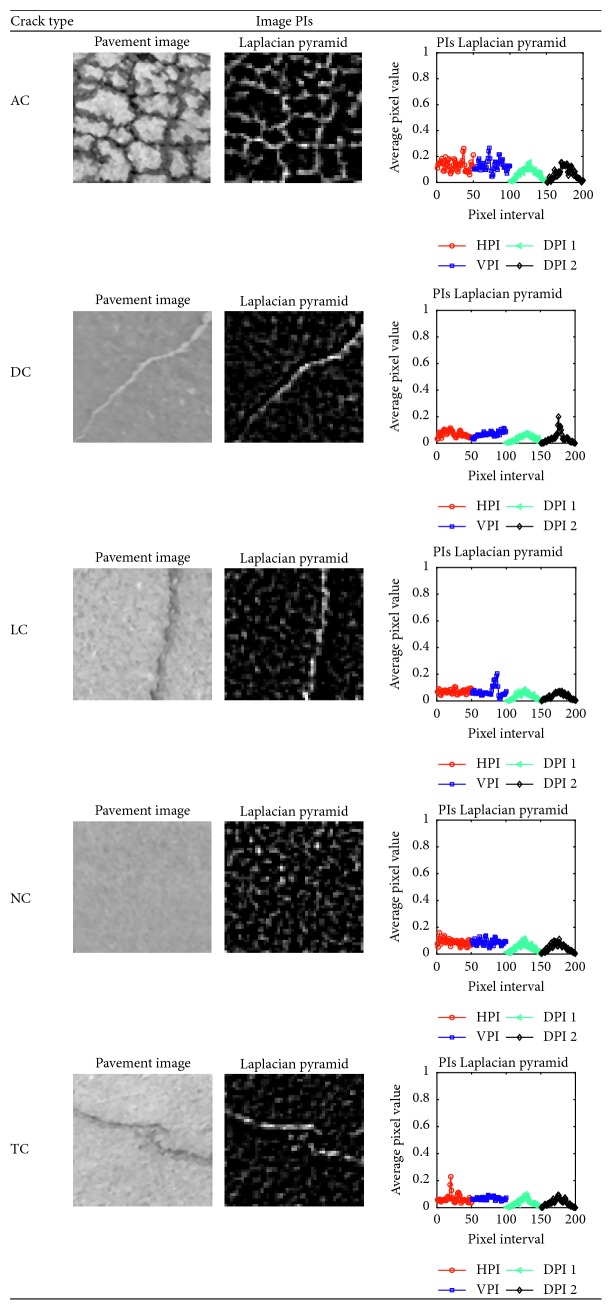
PIs of an image computed by the Laplacian pyramid method.

Besides the two commonly employed HPI and VPI, the diagonal PI (DPI) can also be useful for pavement crack classification, especially in dealing with diagonal cracks. It is proper to note that there are two DPIs in each image. In order to calculate the two DPIs (denoted as DPI1 and DPI2), the output of the Laplacian pyramid operation is rotated with the angles of +45 and −45 to create two rotated maps of salient cracks. The DPI1 and DPI2 are attained by calculating the HPIs of the two rotated maps of salient cracks.  Figure 2 provides illustrations of PIs of pavement images consisting of alligator cracks (AC), diagonal cracks (DC), longitudinal cracks (LC), no cracks (NC), and transverse cracks (TC).

### 2.2. Computational Intelligence Methods

#### 2.2.1. Least Squares Support Vector Machine

LSSVM is a popular tool for pattern classification. First proposed in the previous work of Suykens and Vandewalle [39], this classifier is essentially a least squares version of the standard SVM established by Vapnik [40]. It is noted that LSSVM employs a nonlinear mapping function *φ*(*x*_*k*_) to deal with nonlinear classification problems. The data in the original input space are mapped to a high-dimensional feature space within which a linear classification model can be constructed.

Moreover, it is only necessary to compute the dot product of nonlinear mapping functions during the model construction and prediction phase of LSSVM. The result of this dot product of nonlinear mapping functions is the kernel function *K*(.). Thus, a LSSVM model can be considered as a variant of a neural network model with two layers [41]; the first layer maps the input data into a high-dimensional space and the second layer performs data classification.

LSSVM has demonstrated its superior capability in constructing data-driven models used in various engineering applications [42]. Besides the predictive performance, a notable advantage of LSSVM is its fast training process. It is because the LSSVM model can be trained via a process of solving a system of linear equations instead of the quadratic programming problem required by the standard SVM. The learning phase of LSSVM can be stated in the following constrained optimization problem:

(6) $$\begin{array}{c} \text{minimize}\;J_{p}\left(w,e\right)=\frac{1}{2}w^{T}w+\gamma \frac{1}{2}\overset{N}{\underset{k=1}{\sum }}e_{k}^{2},\\ \text{subjected }\mathrm{to}\;y_{k}\left(w^{T}\varphi \left(x_{k}\right)+b\right)=1-e_{k},\;k=1,...,N, \end{array}$$

where *w* ∈ *R* ^*n*^ denotes a vector perpendicular to the classification hyperplane, *b* ∈ *R* is the bias coefficient and *e*_*k*_ ∈ *R* represents an error variable at the *k*th data instance, *γ* > 0 denotes a regularization parameter which affects the process of penalizing the discrepancy between the observed and the predicted outputs.

The Lagrangian is applied to solve the above constrained optimization problem:

(7) $$\begin{array}{c} L\left(w,b,e;\alpha \right)=J_{p}\left(w,e\right)-\overset{N}{\underset{k=1}{\sum }}\alpha_{k}\left\{y_{k}\left(w^{T}\varphi \left(x_{k}\right)+b\right)-1+e_{k}\right\}, \end{array}$$

where *α*_*k*_ is the Lagrange multiplier and *φ*(*x*_*k*_) denotes a nonlinear mapping function.

Moreover, based on the KKT conditions for optimality, the aforementioned optimization described in Equation (7) can be converted into a linear system [43]. Finally, the LSSVM model used for pattern classification can be compactly written as follows:

(8) $$\begin{array}{c} y\left(x\right)=\mathrm{sign}\left(\overset{N}{\underset{k=1}{\sum }}\left.\alpha_{k}y_{i}K\left(x_{k},x_{l}\right.\right)+b\right), \end{array}$$

where *α*_*k*_ and *b* denote the solutions of the problem described in Equation (7). *K*(*x*_*k*_, *x*_*l*_) represents the kernel function.

The radial-basis function (RBF) kernel is commonly employed; this kernel function is described as follows:

(9) $$\begin{array}{c} K\left(x_{k},x_{l}\right)=\exp\left(-\frac{{\left\|x_{k}-x_{l}\right\|}^{2}}{2\sigma^{2}}\right), \end{array}$$

where *σ* denotes the kernel function parameter which influences the smoothness of the classification boundary.

#### 2.2.2. Differential Flower Pollination (DFP)

As mentioned in the previous section, the model construction phase as well as the prediction phase of a LSSVM model requires the specification of the regularization parameter (*γ*) and the kernel function parameter (*σ*). The problem of model hyperparameter selection, also known as the model selection problem, can be formulated as an optimization problem [44]. Hence, this work employs DFP as a metaheuristic method for optimizing the LSSVM model performance with respect to the two parameters of *γ* and *σ*.

DFP [45] is a population-based metaheuristic method designed for dealing with complex optimization problems in continuous domains. It is noted that the selection of the LSSVM tuning parameters is a complex problem. The first reason is that it is virtually impossible to know the characteristics of the objective function of the optimization problem. The second reason is that the tuning parameters (*γ* and *σ*) are optimized in continuous domains. Hence, there is an infinite number of the candidate solutions. Therefore, DFP is a suitable approach to the problem of the LSSVM model optimization.

Notably, the DFP operation is a combination of two individual metaheuristic methods of the differential evolution (DE) proposed by Storn and Price [46] and the Flower Pollination Algorithm (FPA) proposed by Yang [47]. Thus, DFP inherits the exploitative capability of the DE-based mutation-crossover operators and the explorative capability of the FPA-based global pollination operator. Notably, the Levy flight-based global pollination in FPA significantly helps us to enhance the explorative search by utilizing large step sizes [48, 49].

At the first iteration, all members within the population are randomly generated in the feasible regions. After that, each member modifies its current location through either the global pollination operator or the local pollination operator. According to the suggestion of Yang [47], a selecting probability *p*=0.8 is employed to specify the frequencies of the global and local pollination operations.

The global pollination operator of DFP, inherited from the FPA algorithm, is described in the following equation:

(10) $$\begin{array}{c} X_{i}^{\text{trial}}=X_{i}^{g}+L.\left(X_{i}^{g}-X_{\text{best}}\right), \end{array}$$

where *g* is the index of the current iteration. *X*_*i*_^trial^ denotes a trial solution.

The local pollination process of DFP which is similar to the mutation and crossover of the DE, creates the mutated and crossed solutions as follows:

- Mutated solution:

(11) $$\begin{array}{c} X_{i,g}^{\text{mutated}}=x_{r1,g}+F.\left(x_{r2,g}-x_{r3,g}\right), \end{array}$$

where *r*1, *r*2, and *r*3 denote three random indices used to select three members in the current population. *F* is the parameter of a mutation-scale factor. *F* is generated from a normal distribution with the mean = 0.5 and the standard deviation = 0.15 as suggested in previous works [45, 50].

(ii) Crossed flower:

(12) $$\begin{array}{c} X_{j,i,g+1}^{\text{crossed}}=\left\{\begin{array}{c} X_{j,i}^{\text{mutated}},\;\text{if}\;\;{\mathrm{rand}}_{j}\leq \text{Cr}\;\;\mathrm{or}\;\;j=rnb\left(i\right),\\ X_{j,i,g},\;\text{if }{\mathrm{rand}}_{j}>\text{Cr}\;\;\mathrm{and}\;\;j\neq rnb\left(i\right), \end{array}\right. \end{array}$$

where Cr is the crossover probability which is fixed to be 0.8 as recommended by Price et al. [51].

### 2.3. The Collected Dataset of Pavement Images

Because LSSVM belongs to the category of a supervised learning approach, a dataset of pavement images with ground truth surface conditions must be prepared for training and validating phases. Thus, this research has carried out a survey along several road sections in Hai Chau and Thanh Khe districts, Da Nang city (Vietnam), to collect asphalt pavement images. The images are taken by the human inspector with a digital camera held at the distance of about 1.2 m above the road surface.

**Figure 3:**
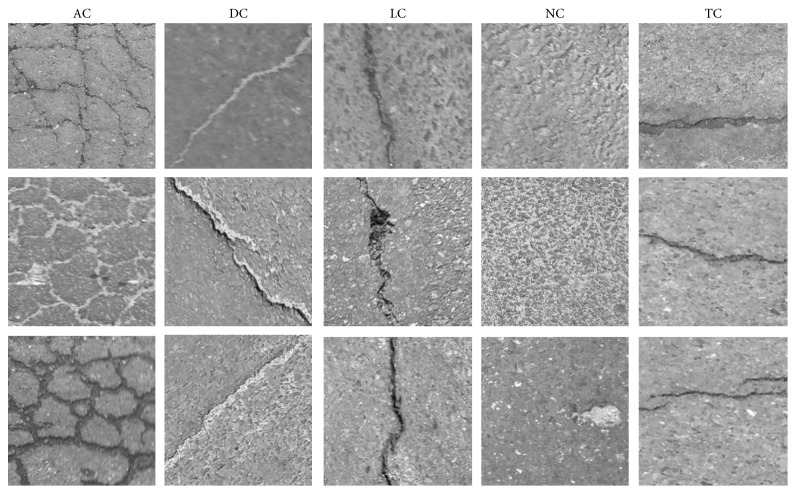
The collected dataset of pavement images.

To accelerate the phases of data processing, feature extraction, and data classification, the size of each image sample has been fixed to be 200 × 200 pixels. Moreover, each image sample is associated with one of the five classes of pavement conditions: alligator crack (AC), diagonal crack (DC), longitudinal crack (LC), noncrack (NC), and transverse crack (TC). It is noted that each individual class of images has 100 samples; thus, the total number of data samples is 500.  Figure 3 illustrates collected image samples. It is noted that the original pavement images have also been enhanced by the widely employed median filter with the window size of 5 × 5 pixels to remove dot noise of the pavement background.

## 3. The Proposed Approach for Classifying Pavement Cracks

**Figure 4:**
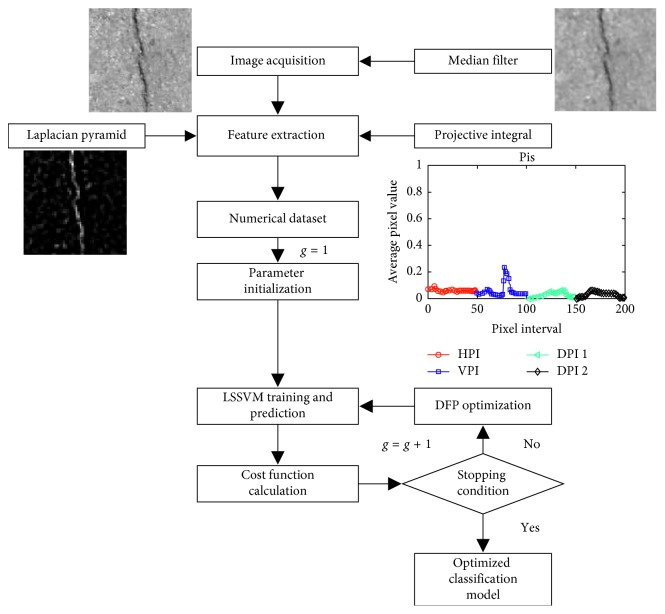
The proposed pavement crack classification model.

This section describes the structure of the proposed model that combines image processing and computational intelligence for pavement crack classification. The model relies on the Laplacian pyramid to generate a salient map of pavement cracks. This map is then processed by the PI technique to create numerical features used by computational intelligence approaches of LSSVM and DFP. As aforementioned, this study resorts to the DFP algorithm to optimize the performance of the LSSVM classification model. In the subsequent parts of the paper, the DFP-optimized LSSVM is denoted as DFP-LSSVM. The overall picture of the proposed model is presented in Figure 4.

It is noted that the proposed pavement classification model is developed in the MATLAB environment with the help of the Image Processing Toolbox [52] and the Statistics and Machine Learning Toolbox [53]. The LSSVM classification model is implemented via the LS-SVMlab Toolbox developed by De Brabanter et al. [54]. The model basically consists of the following two modules:

- Feature extraction, which is based on the Laplacian pyramid and PI techniques
- Data classification based on DFP-LSSVM

**Figure 5:**
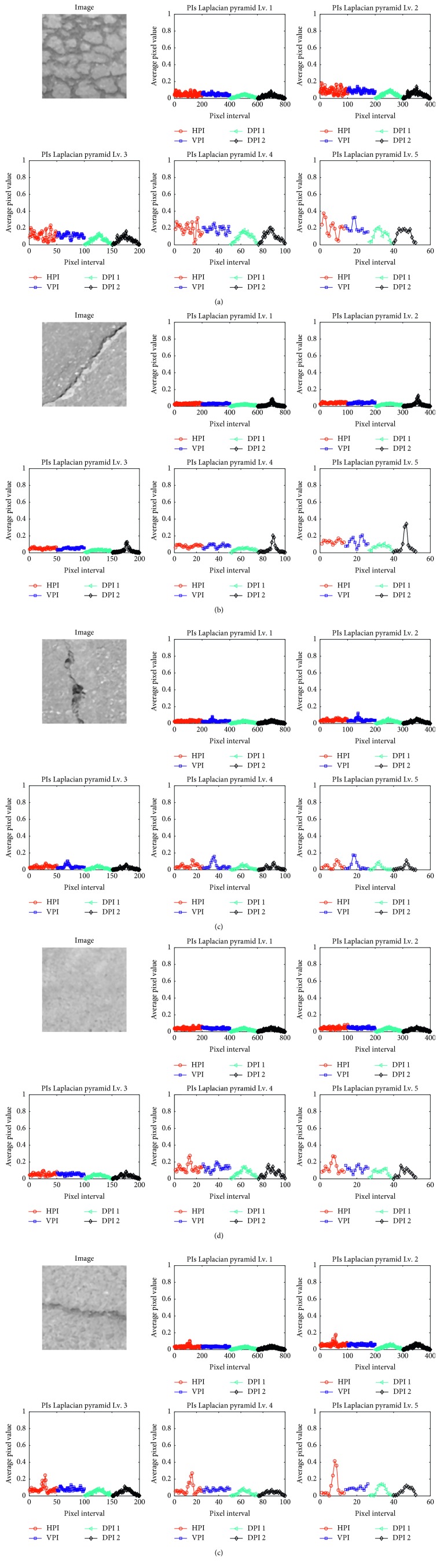
PIs of images corresponding to different Laplacian pyramid levels: (a) AC, (b) DC, (c) LC, (d) NC, and (e) NC.

In the feature extraction phase, pavement images are first processed by the Laplacian pyramid to generate salient crack maps. The PI technique is then employed to compute the four PIs based on such maps. As aforementioned, the image size is 200 × 200 pixels. Therefore, the number of IP-based features created by the first Laplacian pyramid is 200 × 4 = 800. The number of IP-based features created by the next level of the Laplacian pyramid is equal to the number of IP-based features generated by the previous level divided by two. Therefore, the number of features in the second level is 400; the number of features in the third level is 200, and so on.  Figure 5 illustrates PIs of images corresponding to different Laplacian pyramid levels.

After the feature extraction phase, the numerical dataset used for pavement crack classification is constructed. To assess the predictive capability of DFP-LSSVM, the numerical dataset is divided into two different sets: training set (80%) and validating set (20%). In addition, the input variables of the dataset, which are the PI-based features, have been normalized by the *Z*-score transformation. This step aims at standardizing the ranges of variables. The equation of the *Z*-score data transformation is shown as follows:

(13) $$\begin{array}{c} X_{\text{N}}=\frac{X_{\text{O}}-m_{X}}{s_{X}}, \end{array}$$

where *X*_N_ and *X*_O_ represent the normalized and the original features, respectively. *m*_*X*_ and *s*_*X*_ denote the mean value and the standard deviation of the original features, respectively.

Since the performance of LSSVM is strongly influenced by its parameters of *γ* and *σ*, this study relies on DFP to fine-tune these two parameters to achieve the most desired prediction accuracy. At the first iteration (*g*=1), the two hyperparameters (*γ* and *σ*) of LSSVM are randomly created within the feasible regions according to the following equation:

(14) $$\begin{array}{c} \text{Par}=\text{LB}+\text{RN}\times \left(\text{UB}-\text{LB}\right), \end{array}$$

where Par represents the tuning parameter (either *γ* or *σ*) at the first iteration. RN denotes a uniform random number generated within the range of 0 and 1. The LB and UB of *γ* are set to be 0.01 and 1000; the LB and UB of *σ* are selected to be 0.01 and 10.

To identify the most suitable values of *γ* or *σ*, the training set is further divided into subset 1 and subset 2. The first subset is used for model construction, and the second set is used for model validating. These two subsets are only employed for optimizing the LSSVM's parameters. Accordingly, the following objective cost function is used within the DFP algorithm:

(15) $$\begin{array}{c} F_{\text{DFP}}=\frac{\sqrt{\sum_{c=1}^{5}{\left(100-CAR_{c,\text{subset}1}\right)}^{2}}+\sqrt{\sum_{c=1}^{5}{\left(100-CAR_{c,\text{subset}2}\right)}^{2}}}{2}, \end{array}$$

where CAR_*c*_ denotes the classification accuracy rate (CAR) of LSSVM when this classifier predicts data instances in the *c*th class.

CAR used to express the model predictive capability is calculated as follows:

(16) $$\begin{array}{c} \text{CAR}=\frac{N_{D,A}}{N_{D}}, \end{array}$$

where *N*_*D*,*A*_ and *N*_*D*_ denote the number of correctly classified data instances and the total number of data instances, respectively.

The inclusion of prediction results of the subset 1 and subset 2 in the cost function is to guide the population members of DFP to maximize the prediction accuracy of the LSSVM for all the five classes of interest (AC, DC, LC, NC, and TC). The involvement of the subset 2 as validating data is to alleviate model overfitting. Overfitting happens when the LSSVM model classifies the image samples in the training set well, but it classifies the image samples outside the training set incorrectly. Thus, to reduce the effect of this undesired phenomenon, it is beneficial to acquire the LSSVM model that has high CAR in both training set and validating phases. Moreover, it is noted that the LSSVM model has been equipped with the one-versus-one (OvO) strategy [55] to deal with the multilabel data classification. It is because the OvO strategy can help us to achieve good prediction results and also can help us to avoid the problem of imbalanced data classification [56].

During the optimization process, DFP carries out the optimization process until a sufficient number of searching iteration is reached. The maximum number of iteration is fixed to be 100 in this study. When the optimization process terminates, the optimized LSSVM model with the fine-tuned hyperparameters of *γ* and *σ* is ready for classifying the image samples stored in the testing dataset.

## 4. Experimental Results

As stated previously, the dataset including 500 image samples is used to create and verify the performance of the six machine learning models. The dataset is divided into a training set (80%) and a testing set (20%). The first set is employed in the model construction phase; the second set is used to demonstrate the model generalization capability. Since a single run may not reflect the true performance of each machine learning approach due to the randomness in the data selection process, this study repetitively performs the training and testing processes 30 times. The model performance is then evaluated by averaging the outcomes obtained from the 30 times of training and testing data samplings.

To demonstrate the capability of DFP-LSSVM, its performance is compared to those of the classification tree (CTree) [57], linear discriminant analysis (LDA) [58], naïve Bayesian classifier (NBC) [58], and backpropagation artificial neural network (BPANN) [59]. The CTree, LDA, NBC, and BPANN models are implemented in the MATLAB environment via the Statistics and Machine Learning Toolbox [53]. To employ the CTree, LDA, NBC, and BPANN models, it is required to specify their hyperparameters. In this section, the hyperparameters that lead to the best validating performance of models are selected. In the case of the CTree model, the minimal number of observations per tree leaf is chosen to be one as suggested by the MATLAB toolbox [53]. It is proper to note that for guaranteeing a fair comparison, the CTree, NBC, and LDA are also employed with the (OvO) strategy to deal with the multiclass classification problem at hand.

Furthermore, the number of neuron in the hidden layer (denoted as Nr) is an important parameter to be set. Based on the suggestion of Heaton [60], Nr is allowed to vary from 2/3*D* + *O* to 1.5*D* (where *D* denotes the number of input variables and *O* is the number of the output class). In this study, particularly for the datasets constructed from the Laplacian pyramid with levels of 1 and 2, the numbers of features are large. The numbers of features for the Laplacian pyramid with levels of 1 and 2 are 800 and 400, respectively. By several trial and error runs, it is found that the BPANN models with Nr > 300 require long training times and have poor classification performances. Therefore, in these cases, the values of Nr ≤ 300 are investigated to identify the most appropriate BPANN model structure. Moreover, the scaled conjugate gradient algorithm with the maximum number of training epochs = 3000 is employed to construct the BPANN model.

Besides the CAR for each individual class labels, the overall classification accuracy rate (CAR_Overall_) for all the five class labels is calculated by the following equation:

(17) $$\begin{array}{c} {\text{CAR}}_{\text{Overall}}=\overset{5}{\underset{c=1}{\sum }}\frac{{\text{CAR}}_{c}}{5}. \end{array}$$

**Figure 6:**
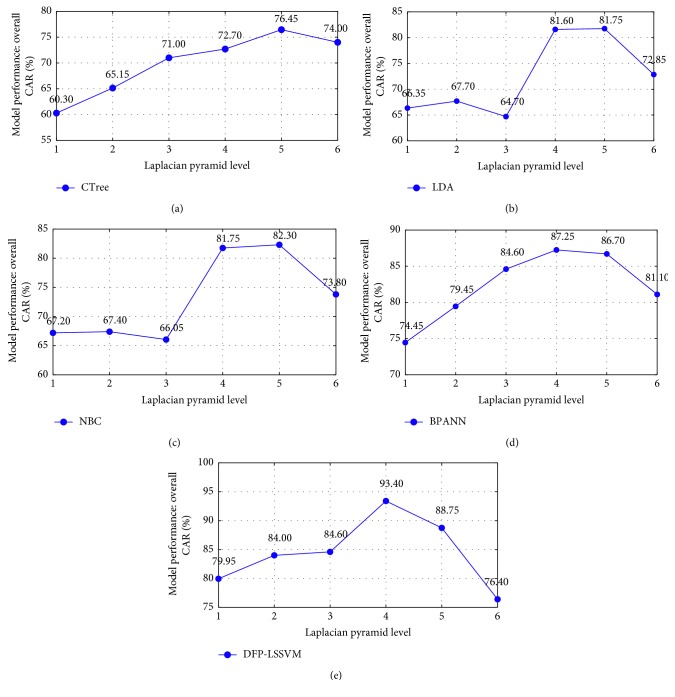
Model performance according to different Laplacian pyramid levels: (a) CTree; (b) LDA; (c) NBC; (d) BPANN; (e) DFP-LSSVM.

Since the Laplacian pyramid is a crucial step in the feature extraction phase, this study has investigated the performances of DFP-LSSVM and other benchmark models according to different levels of the Laplacian pyramid. It is noted that the Laplacian pyramid does not only highlight the crack patterns by creating a salient crack map but also serves as a means of dimension reduction. It is because this technique helps us to reduce the image size and leads to fewer PI-based features. The outcomes of experiments are graphically presented in Figure 6. It can be seen that the increase in the Laplacian pyramid's level imposes positive effects on the model performances. Evidently, CTree, LDA, and NBC achieve the highest overall CAR when the Laplacian pyramid's level = 5. The BPANN and DFP-LSSVM attain the best overall CAR when the Laplacian pyramid's level = 4. A notable observation is that when the Laplacian pyramid's level >5, the model performances significantly drop. It is an understandable phenomenon because high level of the Laplacian pyramid leads to lower image resolutions and causes significant losses of information. If the Laplacian pyramid's level = 6, the information presented in the images is not sufficient for the machine to construct classifiers capable of making accuracy crack pattern recognition.

The details of the model prediction results obtained from the repeated random subsampling process with 20 runs are summarized in Table 1. The CAR of all individual classes (AC, DC, LC, NC, and TC) and the overall CAR are reported with their mean and standard deviation values. It can be observed that DFP-LSSVM has achieved the highest overall CAR of 93.40%, followed by BPANN (87.25%), NBC (82.30%), LDA (81.75%), and CTree (76.45%). The hybrid computational intelligence model of DFP-LSSVM also has the most accurate outcomes in each individual class of AC (91.50%), DC (97.50%), LC (99.25%), NC (81.00%), and TC (97.75%).

**Figure 7:**
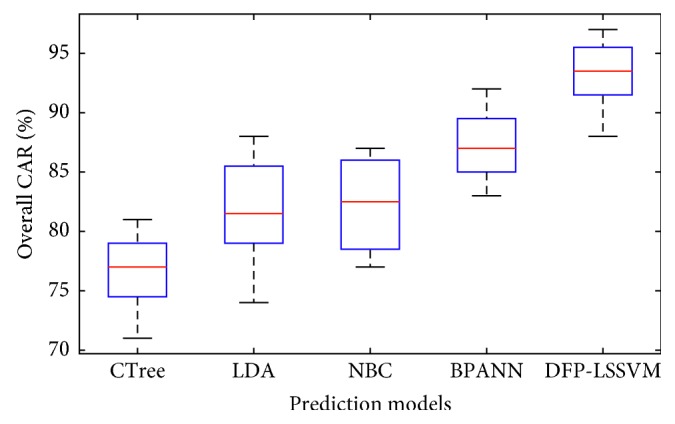
Box plots of model performances.

In addition, Figure 7 presents the box plots of prediction results of the DFP-LSSVM, BPANN, NBC, LDA, and CTree. To confirm the statistical difference of the model performances, this study relies on the Wilcoxon signed-rank test (WSRT). WSRT is a popular nonparametric statistical hypothesis test which is often used for result comparison [61]. In this study, the significance level of WSRT is chosen to be 0.05. If the *p* value computed from the test is smaller than 0.05, it is able to confirm that the pavement crack classification results of the employed models are statistically different. The results of WSRTs are reported in Table 2. With *p* value < 0.05, it is confident to state that the performance of DFP-LSSVM is significantly better than those of other benchmark models.

**Figure 8:**
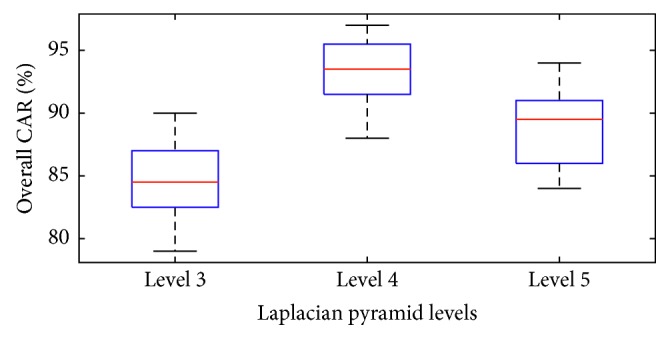
Box plots of DFP-LSSVM model performances with different levels of Laplacian pyramid.

In addition, to confirm that the Laplacian pyramid at the level of 4 is the most appropriate level for the feature extraction phase of the DFP-LSSVM model, and the prediction outcomes of the levels of 3, 4, and 5 are compared. The results of the repeated subsampling obtained from these three levels are illustrated by the box plots in Figure 8. WSRT is also used to inspect the statistical significance of the model performances. The results of WSRT are reported in Table 3. Observably, the *p* values computed from the test indicate that the Laplacian pyramid at the level of 4 is significantly better than that at the levels of 3 and 5. Based on the experimental results and the employed statistical test, the DFP-LSSVM that uses the Laplacian pyramid at the level of 4 deems best suited for the collected dataset at hand.

Hence, the overall finding of this study is that the Laplacian pyramid can be an effective method for achieving a high CAR of pavement crack detection and categorization. The model accuracy of 93.40% is significantly better than the previously constructed models that employ the steerable filtering method. The steerable filtering-based models proposed in [22] and [25] achieved the average CAR of 80.25% and 87.50%, respectively. The model based on steerable filters and support vector machine described in [62] demonstrated good predictive performance (CAR is about 96%); however, this model was incapable of recognizing diagonal cracks. Image processing-based crack recognition approaches based on Gabor filter [11] and steerable matched filtering [15] also demonstrated promising results with the precision being up to 95% and 93%, respectively; however, this approach is only appropriate for assigning crack or noncrack labels to image samples.

A novel method based on the beamlet transform technique has been proposed in [9]; nevertheless, this method was unable to recognize diagonal and alligator cracks. Moreover, intelligent models based on convolution neural networks (CNNs) established in [17, 18, 63] have significant advantages over the models based on image processing algorithms (e.g., Laplacian pyramid and steerable filters); that is, the feature extraction and data classification can be integrated and performed autonomously. These models based on CNNs also attained positive classification accuracy which can be up to 94%; however, they have rarely been performed in multiclass pavement recognition. In the aspect of data classification based on machine learning, the previous works [20, 64–66] have pointed out the appropriateness of neural networks for pavement crack detection. However, this current study has experimentally shown that LSSVM can be more capable than neural networks in the task of interest.

## 5. Conclusion

This study constructs an image processing-based method for classifying pavement crack patterns. The Laplacian pyramid and the PI technique are used to extract numerical features from pavement images. The Laplacian pyramid generates a salient crack map which highlights the patterns of cracks. The PI technique produces the features of the four PIs including HPI, VPI, and two DPIs. These PIs are specifically designed to recognize the four patterns of cracks (AC, DC, LC, and TC) as well as the state of intact pavement (NC). A prediction model that combines the LSSVM and DFP algorithms has been constructed. LSSVM is used to generalize classification boundaries that separate the learning space into five subspaces of AC, DC, LC, NC, and TC. DFP acts as an optimization tool for assisting the model construction phase of LSSVM.

A dataset of 500 images has been collected to train and test the proposed methods of feature extraction and classification. Notably, since the image size processed by the Laplacian pyramid is reduced after each level of analysis, the Laplacian pyramid also helps us to alleviate the computational burden of DFP-LSSVM by decreasing the size of the feature set. Experiments relied on a random subsampling process, and WSRT confirms that DFP-LSSVM used with the Laplacian pyramid at the level 4 can deliver the most desired prediction outcome with the overall CAR = 93.4%. Thus, the newly established model can be a promising tool to ease the labor-intensive periodic pavement survey. The future development of the current work may include the employment of other advanced image processing techniques in the tasks of crack identification. Additionally, comparing different methods used for crack analysis and feature extraction can also be a worth investigating research direction. It is also desirable to investigate other effective classifiers in the task of crack pattern recognition as well as to expand the current dataset by collected more types of cracks (e.g., reflective cracks).

## Figures and Tables

**Table 1:** Prediction result comparison.

Models	Mean of CAR (%)						Standard deviation (std) of CAR (%)					
	AC	DC	LC	NC	TC	Overall	AC	DC	LC	NC	TC	Overall
CTree	78.00	70.75	88.00	62.00	83.50	76.45	9.09	10.17	8.65	9.92	7.80	2.98
LDA	78.75	81.50	96.75	58.75	93.00	81.75	9.72	7.45	4.94	14.04	6.37	4.13
NBC	78.75	83.00	96.50	59.25	94.00	82.30	7.93	6.37	6.51	11.04	4.76	3.51
BPANN	80.75	94.75	95.50	72.25	93.00	87.25	9.36	4.99	5.36	9.52	5.23	2.61
DFP-LSSVM	91.50	97.50	99.25	81.00	97.75	93.40	6.51	3.03	1.83	10.59	3.43	2.62

**Table 2:** Model result comparison *p* values of WSRT.

	CTree	LDA	NBC	BPANN	DFP-LSSVM
CTree	—	0.0007	0.0004	0.0001	0.0001
LDA	0.0007	—	0.7016	0.0012	0.0001
NBC	0.0004	0.7016	—	0.0004	0.0001
BPANN	0.0001	0.0012	0.0004	—	0.0002
DFP-LSSVM	0.0001	0.0001	0.0001	0.0002	—

**Table 3:** *p* values of WSRT (the DFP-LSSVM model).

	Level 3	Level 4	Level 5
Level 3	0.0000	0.0001	0.0003
Level 4	0.0001	0.0000	0.0005
Level 5	0.0003	0.0005	0.0000

## Data Availability

The data used to support the findings of this study are available from the corresponding author upon request.

## Supplementary Materials

Supplementary Materials The supplementary file contains the dataset used in this study. In this file, the first 100 columns are the input features of the data (which are the projection integrals); the last column is the class labels (1 = alligator crack, 2 = diagonal crack, 3 = longitudinal crack, 4 = noncrack, and 5 = transverse crack).
